# Complex implementation mechanisms in primary care: do physicians’ beliefs about the effectiveness of innovation play a mediating role? Applying a realist inquiry and structural equation modeling approach in a formative evaluation study

**DOI:** 10.1186/s12875-023-02081-x

**Published:** 2023-06-27

**Authors:** Sara Söling, Ibrahim Demirer, Juliane Köberlein-Neu, Kira Isabel Hower, Beate Sigrid Müller, Holger Pfaff, Ute Karbach, AdAM Study Group

**Affiliations:** 1 Institute of Medical Sociology, Health Services Research, and Rehabilitation Sciences, Faculty of Human Sciences & Faculty of Medicine and University Hospital Cologne, University of Cologne, Cologne, Germany; 2 Center for Health Economics and Health Services Research, Schumpeter School of Business and Economics, University of Wuppertal, Wuppertal, Germany; 3 Institute for General Medicine, Faculty of Medicine, University Hospital Cologne, University of Cologne, Cologne, Germany

**Keywords:** Digital technology, Polypharmacy, Primary health care, Clinical reasoning, Diffusion of innovation, Behavior and behavior mechanisms, Realist, Evaluation methodology

## Abstract

**Background:**

The adoption of digital health technologies can improve the quality of care for polypharmacy patients, if the underlying complex implementation mechanisms are better understood. Context effects play a critical role in relation to implementation mechanisms. In primary care research, evidence on the effects of context in the adoption of digital innovation for polypharmacy management is lacking.

**Study aim:**

This study aims to identify contextual factors relevant to physician behavior and how they might mediate the adoption process.

**Methods:**

The physicians who participated in this formative evaluation study (*n* = 218) were part of the intervention group in a cluster-randomized controlled trial (AdAM). The intervention group implemented a digital innovation for clinical decision making in polypharmacy. A three-step methodological approach was used: (1) a realist inquiry approach, which involves the description of a context-mechanism-outcome configuration for the primary care setting; (2) a belief elicitation approach, which involves qualitative content analysis and the development of a quantitative latent contextualized scale; and (3) a mediation analysis using structural equation modeling (SEM) based on quantitative survey data from physicians to assess the mediating role of the contextualized scale (*n* = 179).

**Results:**

The key dimensions of a (1) context-mechanism-outcome model were mapped and refined. A (2) latent construct of the physicians’ innovation beliefs related to the effectiveness of polypharmacy management practices was identified. Innovation beliefs play a (3) mediating role between the organizational readiness to implement change (*p* < 0.01) and the desired behavioral intent of physicians to adopt digital innovation (*p* < 0.01; *R*^2^ = 0.645). Our contextualized model estimated significant mediation, with a relative size of 38% for the mediation effect. Overall, the model demonstrated good fit indices (CFI = 0.985, RMSEA = 0.034).

**Conclusion:**

Physician adoption is directly affected by the readiness of primary care organizations for the implementation of change. In addition, the mediation analysis revealed that this relationship is indirectly influenced by primary care physicians’ beliefs regarding the effectiveness of digital innovation. Both individual physician beliefs and practice organizational capacity could be equally prioritized in developing implementation strategies. The methodological approach used is suitable for the evaluation of complex implementation mechanisms. It has been proven to be an advantageous approach for formative evaluation.

**Trial registration:**

NCT03430336. First registration: 12/02/2018. ClinicalTrials.gov.

**Supplementary Information:**

The online version contains supplementary material available at 10.1186/s12875-023-02081-x.

## Background

The implementation of digital health technologies is expected to improve the quality of care and simplify clinical actions [1]. As an additional outcome, there is evidence that the implementation of digital systems, such as clinical decision support systems, can have a positive impact on patient safety [2–4]. Despite the potential to improve patient outcomes, research on implementation often demonstrates inconsistent effectiveness. This may be related to proximal outcomes of the actual complex implementation behavior during the change processes [5, 6]. The effectiveness of implementing and adopting digital innovations varies considerably across healthcare organizations [7]. Therefore, the underlying mechanisms are of great interest.

Research has already been conducted on a number of technology-related factors, including the interoperability of new technologies with existing practice systems [8], the availability of information on screen versus on paper [6], the compatibility of health IT and clinical work processes [9], and the level of physician involvement in the development of new technologies [5]. However, there is still limited evidence to date on the relationship between the context in which a digital innovation is implemented and its effectiveness [2, 6].

As part of the digital transformation of the German healthcare system, we sought to understand the complex implementation mechanisms that lead to the adoption of a digital innovation for polypharmacy management. Therefore, this formative evaluation study within the cluster-randomized controlled trial (cRCT) project “Application of a Digitally Assisted Pharmacotherapy Management System” (AdAM project—original German acronym for the project), was designed to analyze individual and organizational contextual factors related to implementation mechanisms [10]. A qualitatively described configurational model of context, mechanism, and outcome was developed for our study setting, and the intersection with structural equation modeling (SEM) was explored [11, 12]. In keeping with the realist approach, our study aimed to better understand the effects of contextual features on the implementation behavior of primary care physicians.

Our research questions were the following:

- What are the possible implementation mechanisms by which digital innovation for polypharmacy management results in intended outcomes?
- How and in what context does the digital innovation work for primary care physicians?

### Theoretical framework

Although it is important to identify influential technology-related factors to analyze the complex implementation mechanisms of a digital innovation, these factors were not sufficient as explanatory variables for our theoretical framework. In our study design, we define complexity in terms of both the different levels of the social system in which an innovation is implemented and the influences of the context itself. In particular, the interaction of these factors in implementation mechanisms can be considered complex and the results unpredictable.

Therefore, disaggregating the different levels of the social system of primary care organizations was an important prerequisite to make the study of the complexity of implementation mechanisms more manageable for data analysis. In a subsequent step, the disaggregation enabled us to analyze how the inhibiting or facilitating contextual factors at the different levels influence adoption [13]. In particular, at the meso-organizational and micro-behavioral levels, the empirically studied and known implementation factors may have influenced the social system responses of primary care practices [14, 15].

### Barriers and facilitators to adoption

Empirically studied implementation barriers and facilitators found in the initial literature search were categorized as follows:

1. Meso level: Research on organizational determinants that affect adoption include numerous topics, such as organizational culture; organizational readiness for change; networks and communication (collaboration and teamwork); resources (financial, education, and training); and leadership [16–20].
2. Micro level: The technology acceptance model (TAM) has been widely used as a research model since the 1980s to study the behavior-related micro-level determinants of adoption during the IT implementation process [21]. TAM is based on an adapted version of the social-cognitive theories of reasoned action (TRA) and planned behavior (TPB). The technology acceptance model measures dimensions of technology-related behavioral aspects, such as ease of use and usefulness, that influence the stakeholders’ intentions to use [22]. In particular, the construct of behavioral intentions to use innovation—as the most proximal antecedent to actual information technology (IT) use—intersects with the outcome adoption of the implementation. Intention has been used synonymously in numerous studies to measure adoption [23] and has been demonstrated to be a valid proxy measure for the behavior of physicians [24]. On average, intention can account for 28% of the variance in behavior [25]. The behavioral factors described above, namely intention to use an innovation or adoption of an innovation, are particularly important for the formative evaluation of implementation processes. Contextual behavior-related factors may explain observed variation in implementation effectiveness or influence how clinicians cope with implementation challenges and how they interact with innovation in the health IT adoption process [26, 27].

### Context in implementation research: a new approach

In addition to the empirically observed organizational and behavioral determinants, we examined the current state of research on context in implementation research [10, 28, 29]. Implementation research has shown that certain clinical practices are complex in nature (for example, the prescription of multiple drugs). Moreover, the adoption of new and complex practices may be influenced by facilitating or inhibiting contextual features.

Context is defined as “the relational and dynamic features that shape the mechanisms through which the intervention operates; context is assumed to be dynamic and emerge over time at several different levels of the social system” [10, 28]. From an implementation research perspective, empirical research should not only focus on the targeted clinical practice, but also on the contextual features of the implementation. The success of an innovation is inseparably linked to the context in which it is implemented [21].

Advanced empirical research is needed to understand the unresolved causal relationships between the contextual characteristics of implementation and the adoption of new and complex practices [16]. Although implementation science has developed several conceptual models to address contextual complexity [28, 29], these models lack specific methods for conducting empirical research. These findings emphasize the importance of developing new methods of analysis to determine the impact of contextual features on the complex responses of adaptive primary care social systems for three reasons: (1) to explain the emergence of generative mechanisms in implementation, (2) to explain the differences in implementation outcomes, and (3) to be able to develop targeted implementation strategies based on the discovered underlying mechanisms [11].

For this purpose, already confirmed general concepts from implementation and complexity research, health services research, and technology acceptance research can be integrated to generate empirically testable research models [17, 22, 29]. However, research models must also incorporate data-driven concepts adapted to the specific study context to gain new insights and to capture the complexity of context in implementation. In relation to our study objective, we sought to address the above challenges and to apply a novel methodological approach to an example of implementing digital innovation to manage polypharmacy in primary care (see Additional File 1).

## Methods

Thus, the paradigm of context in (1) realist approaches is situated scientifically and analytically between positivist and constructivist approaches. The goal is to discover semi-predictable patterns related to contexts, underlying generative mechanisms, and outcomes (CMO), and to develop middle-range theories related to the object of study [10]. We used the formula revised by Dalkin et al. (2015), according to which a mechanism includes both resources and reasons. In addition, we assume a strong connection between context and reasoning (mechanism [resources] + context → mechanism [reasoning] = outcome) [11]. The revised formula suggests an alternative operationalization of context in realist approaches in which “intervention resources are introduced in a context, in a way that enhances a change in reasoning. This alters the behaviour of participants, which leads to outcomes.” In this process, we used both the findings from an initial literature review and the results of a qualitative data analysis. The choice of the description of a context-mechanism-outcome configuration for the study setting was the result of an abductive synthesis process. In addition, the conclusions drawn from that description were used to operationalize the structural equation model.

We then used the (2) belief elicitation approach to develop a contextualized latent scale. This data-driven approach allows the contextualization of behavior-related assumptions for a particular setting, population, or new behavior of interest [19, 22]. In technology acceptance research, the belief elicitation approach is recommended to identify health-related variables from the participants’ perspective instead of arbitrarily including variables [22].

Regarding our research questions, we assumed that the (3) structural relations between meso and micro levels in participating primary care organizations should be differentiated in the structural equation model. Two objectives were pursued for empirical investigation: (a) to explain the influence of an organization-related variable on the implementation mechanism and the outcome of implementation (adoption) and (b) to examine the mediating effect of physicians’ contextualized innovation beliefs. Relevant constructs were operationalized for different levels of the organization to enable the mediation model to explain the complex implementation pathways. Testable hypotheses were generated based on the different methodological and analytical steps applied.

### Data collection and research design

We collected qualitative data (from May to September 2018) and quantitative data (from November 2019 to January 2020) from primary care physicians who participated in the formative evaluation study of the AdAM project. This formative evaluation study was conducted alongside the stepped-wedge, cRCT in AdAM. In the cRCT study protocol, we described the study design of our formative evaluation study, in which we aimed to examine physician-side barriers and facilitators to the implementation process using a mixed methods approach (see Additional File 2) [30].

Interviews with physicians from the intervention group were conducted to determine their experiences with digital innovation (see Additional File 3). Focus groups were conducted with both arms of the RCT. The objective was to compare project-related expectations and experiences according to the participants’ cRCT group. All interviews and focus groups were conducted or moderated by the first author of this article (SS). Data were independently coded by two researchers from the University of Cologne’s research team. MAXQDA was used to support data coding.

Data from the cross-sectional survey of physicians were used for structural equation modeling. The sample for the survey included all the physicians in the intervention group, who had enrolled at least one patient in the study. To increase the response rate, we used the tailored design approach by Dillman, which means that the physicians were reminded three times to respond to a questionnaire administered by post [31].

We used a sequential and exploratory design. Qualitative data analysis was conducted in an exploratory manner in the first phase of the study to identify categories related to the range of physician expectations and experiences, which were then used in the second phase of the study to develop a quantitative measurement instrument and build a model. In addition, we triangulated the data at the modeling level as the qualitatively developed model was transformed into a quantitative model. The results of the first phase of the study were confirmed in the second phase of the study in an attempt to reduce bias in the interpretation of the results. A meta-inference was generated by merging the inferences from the CMO and the mediation model to provide the final description of the mechanism [32, 33].

### Description of the innovation

The digital innovation was implemented in 688 recruited general practices in North Rhine-Westphalia. It was expected to improve prescription quality and safety for adult patients with polypharmacy compared to patients receiving standard care. The innovation included several design components (e.g., a digitalized clinical decision support system for polypharmacy, patient medication history and diagnosis, information about other specialists, training on system use and management, technical support for physicians, and recommendations for prescribing in polypharmacy).

### Data analysis

Qualitative data analysis was conducted for two purposes: (1) summarizing content-deductive mapping of the data material with the aim of describing the categories of context, resources, reasoning, and outcome (CMO) and (2) application of the belief elicitation approach through deductive-inductive qualitative content analysis to develop the latent measurement tool of contextual innovation beliefs for the structural equation model (SEM). A content analysis approach was adopted, which incorporated elements of conventional and directed content analysis [34]: conventional because interview data were used to describe the range of physicians’ responses to innovation, and directed because the guides for interviews and focus groups were thematically structured and theory-driven, based on the results of the literature review. The content analysis was conducted in a deductive-inductive fashion: it was deductively oriented to the categories of CMO and the interview guides and inductively derived categories from the data material.

The quantitative items were operationalized based on categories identified by the content analysis and inserted into the structural equation model as latent variables with their measurable indicators (*n* = 179). The validity and reliability of the final construct were tested through factor analysis in the structural equation measurement model. We decided to apply SEM because it is particularly useful for mediation analysis and testing relationships between latent constructs, which otherwise remain unobserved or cannot be directly assessed [35, 36]. Structural equation modeling facilitates the analysis of latent constructs through the observed indicators representing the constructs of interest and provides multivariate evidence of causal mechanisms.

A two-stage approach to quantitative data analysis was implemented. In this approach, the measurement model and the structural model were analyzed separately [37]. The measurement model specifies the relationships between latent constructs and observed measures, whereas the structural model specifies the relationships among latent constructs and includes multivariate regression models [35]. The models were analyzed using STATA 15.1, and graphical path models were created. A covariance matrix was utilized as an input, and maximum likelihood with bootstrapped estimators was generated (200 replications). No missing values were inputted. The quality of the measurement model was tested by confirmatory factor analysis (CFA). Convergent validity was established by examining the significance of individual item loadings.

We evaluated model fit using the comparative fit index (CFI) and the Tucker–Lewis index (TLI). The values of CFI and TLI range from 0 to 1, with values from ≥ 0.90 to ≥ 0.95 representing acceptable to good fit [36]. In addition, we examined the root mean square error of approximation (RMSEA) and the associated confidence interval and *p* value. We considered RMSEA values < 0.08 and an upper bound of the confidence interval < 0.1 to be acceptable [38]. Furthermore, we assessed discriminant validity by comparing the average variance extracted for each construct to the squared correlation between two latent variables at one time point. Estimations of composite reliability and average variance extracted, as well as investigations of internal reliability (Cronbach’s alpha), were the last steps of the measurement model analysis.

### Hypothesis development: structural equation model

**Fig. 1:**
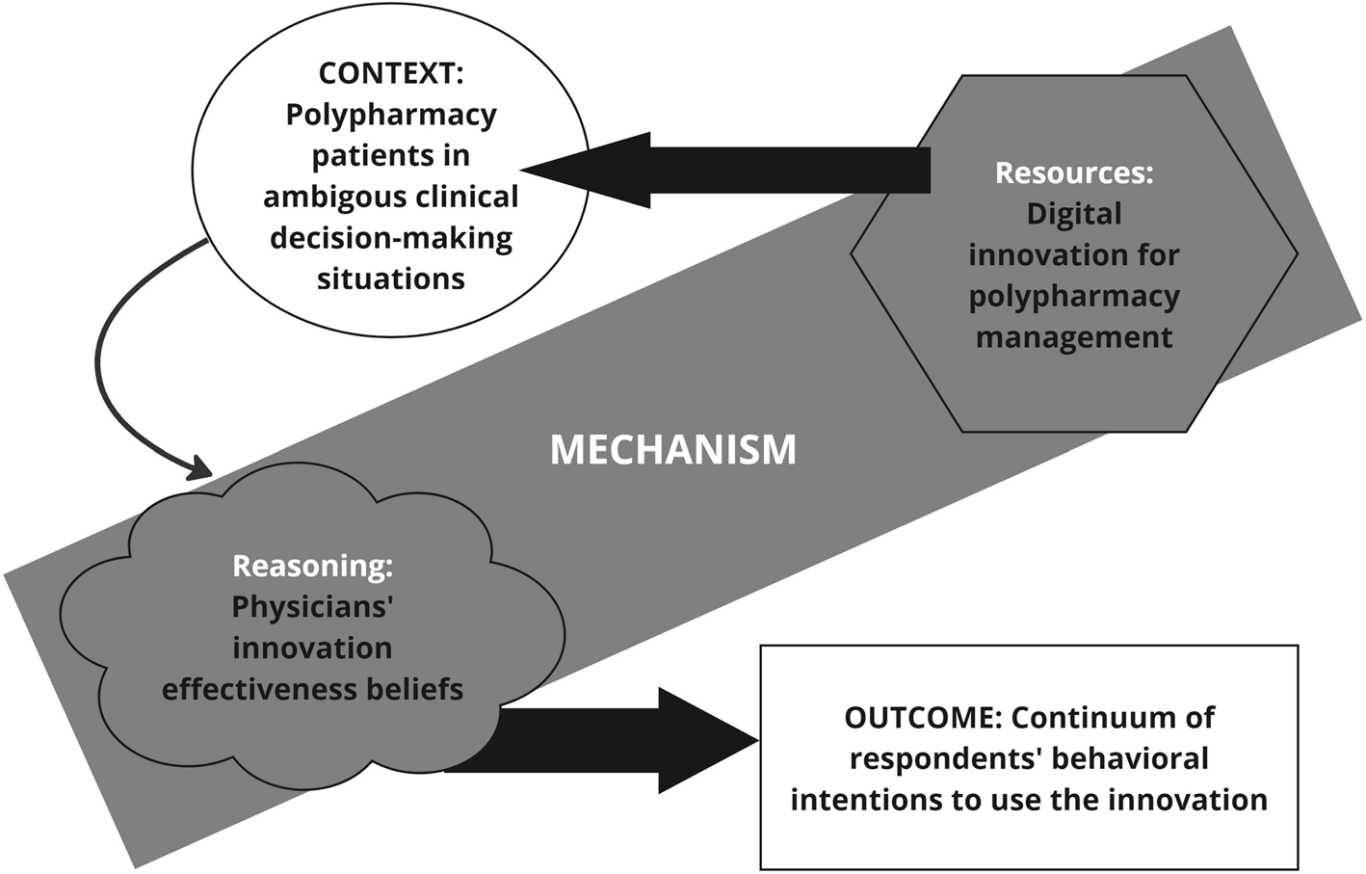
Context-mechanism-outcome model for physician delivery of digital innovation

**Fig. 2:**
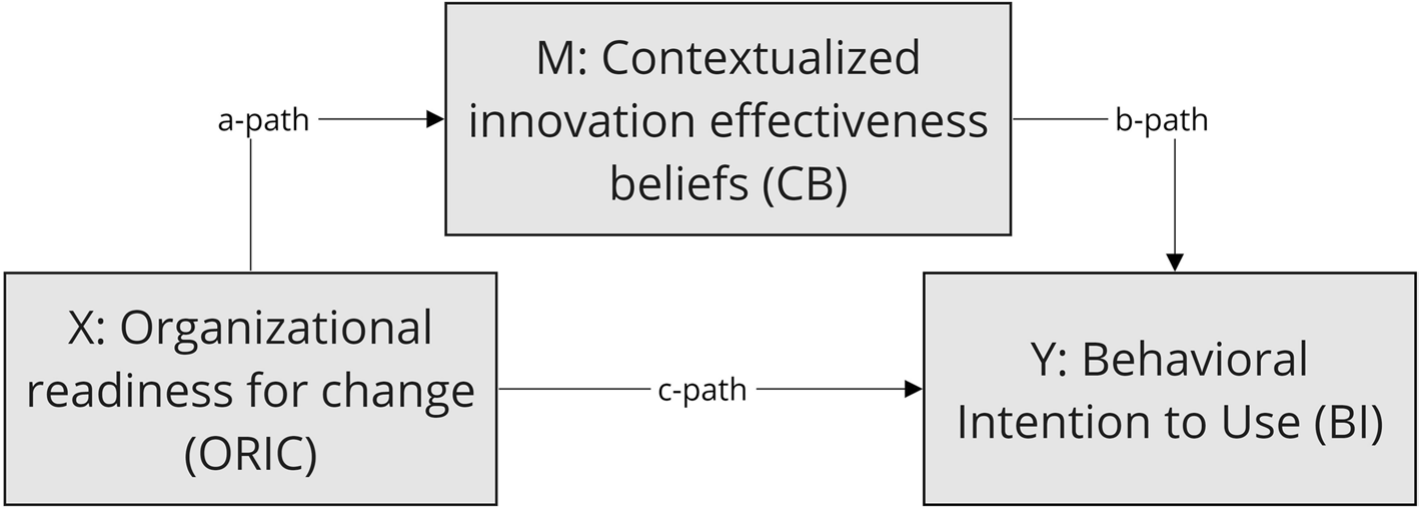
Mediation model Note: X = independent variable, M = mediator, and Y = outcome. The indirect effect is estimated as the product of the a- and b-paths (i.e., a*b). The c-path represents the direct effect of X on Y (i.e., the effect of X on Y that is not transmitted through the mediator, M)

Because structural equation modeling focuses on testing of models of hypothesized theoretical relationships, we synthesized the results of our literature review to select theory-based latent constructs with the findings related to the qualitative configurational model (see Fig. 1) and integrated the data-driven construct developed to build the structural equation model (see Fig. 2).

We chose the construct of organizational readiness for change as an organizational variable at the meso level [17, 18, 39]. Organizational readiness models provide a perspective on the extent to which members of an organization are psychologically and behaviorally prepared to implement organizational change. Technology acceptance models (TAMs) were selected as a micro level theoretical approach. TAMs focus on attitudes and behavioral intentions to use health IT [22]. With the use of a TAM, we included the intentions/adoption construct in the research, but none of the other constructs that measure other technology-related aspects. Regarding health IT adoption, alternative models suggest that organizational readiness to change can influence the individual level and, thus, the actual usage and adoption [17].

At the meso level, we hypothesized that organizational readiness for change is an important prerequisite to enable physicians to evaluate the effectiveness of innovation (mechanism [reasoning]; meso- and micro-level relations; a-path). This assumption was based on what our previous qualitative analyses demonstrated. For example, the process of patient registration and the groundwork for transferring additional patient data into digital innovation depend on the readiness of the primary care employees. They have to adapt to these changes brought about by the implementation of innovation. Both these tasks are related to organizational readiness and are important prerequisites for activating the part of the mechanism that corresponds to physician reasoning. The primary role of the physician is to make appropriate clinical decisions regarding the prescription of medications for patients with polypharmacy. Contextualized beliefs regarding the effectiveness of innovation consist of several components. These components are important from the pragmatic perspective of physicians in the management of polypharmacy patients in ambiguous clinical decision situations (context → mechanism [reasoning]). We hypothesized that physicians would only perceive the innovation as being effective if it addressed components relevant to the care of polypharmacy patients on a practical level.

Moreover, physicians’ strong contextual beliefs increase a positive effect on the intention to use the innovation (b-path). Finally, we assessed whether the hypothesized direct relationship between the organization’s readiness to implement innovation and the physician’s intention to adopt (c-path) is mediated by the physicians’ belief in the effectiveness of innovation.

Our empirical research questions for the mediation analysis are as follows: Do physicians’ contextualized beliefs regarding the effectiveness of innovation (micro level) mediate the relationship between primary care organizations’ readiness for change (meso level) and physicians’ adoption behavior (micro level) during the change process of implementing digital innovation for polypharmacy management? How strong are the direct and indirect effects?

Based on the previous discussion, the following hypotheses are proposed:

- H1: Organizational readiness for change has a positive direct effect on behavioral intention to use digital innovation (c-path).
- H2: Organizational readiness for change has a positive direct effect on physicians’ contextualized innovation effectiveness beliefs (a-path).
- H3: Physicians’ contextualized innovation effectiveness beliefs have a positive direct effect on behavioral intention to use digital innovation (b-path).
- H4: Physicians’ contextualized innovation effectiveness beliefs mediate the relations between organizational readiness for change and behavioral intention to use digital innovation.
- H5: Physician and structural characteristics have direct and indirect effects on contextualized innovation beliefs and intention to use digital innovation.

### Measurement instrument

The data collected in our survey were used to develop the SEM model. The questionnaire was pre-tested in two stages: in think-aloud interviews (*n* = 4) to assess the comprehensibility of the questions and in a postal sample (*n* = 10) to determine whether the filter guidance worked and whether the range of the scales was used. The findings were used to modify the questionnaire into the final version and improve its quality.

Accordingly, the survey included measurement items for each of the models’ latent constructs: ORIC (organizational readiness for implementing change) [39], CBs (contextualized innovation effectiveness beliefs), and BI (behavioral intention to use/adoption) [40]. Each of the constructs was measured using multi-item scales.

Physicians answered the organization-related aspects of our questionnaire as key persons of the participating practice. Measurement based on individuals’ assessments of collective capabilities is preferable when collective outcomes depend on skillful teamwork [41]. The organization-related instrument ORIC and individual-related instruments CBs and BI had an adequate item structure (items were written from the perspective of the collective for the organization-related instrument and from the perspective of the individual for individual-related instruments). For all measurement instruments, data were not aggregated at the cluster level because nearly all physicians in our sample were solo practice owners and did not work in group practices (cluster level).

### Measurements: organizational readiness for implementing change (ORIC) (X)

The nine items used to measure ORIC are adapted from Shea et al. and the validated German version [39, 42]. The scale measures the extent to which members in an organization are psychologically and behaviorally prepared to implement organizational change (e.g., with items such as “People who work here are committed to implementing contents of the AdAM project” or “Challenges may arise in the implementation of the contents of the AdAM project. The people who work here are confident that they can overcome them”) [42].

### Measurements: contextualized innovation effectiveness beliefs (mediator)

Following the realist research approach, the items of the CB construct were developed to measure different components of beliefs regarding the effectiveness of innovation. The empirically observed influence of the context on the reasoning process from the qualitative analyses was included in the scale. The construct includes six items related to three components of physicians’ contextualized beliefs related to polypharmacy management practices: (1) *safety of the prescription*: the perceived increase in awareness of prescription risks of polypharmacy and the transfer of newly gained knowledge about multimedication to other patients in the medical practice; (2) *information quality:* the perceived increase in information quality related to polypharmacy risk and adverse effect analyses; and (3) *communication:* the perceived improvement in doctor–patient communication (Cronbach’s α = 0.86) (see Additional File 4).

### Measurements: behavioral intention to use (BI) (Y)

Three items are used to measure the behavioral intention to use technology for routinely performed and future work tasks (“I routinely use digital innovation for my work with polypharmacy patients,” “I would like to continue to use digital innovation for my work,” “I have performed many of the routine tasks for my polypharmacy patients with the help of digital innovation”). In particular, we used a BI scale, which was validated, translated into German, and checked for reliability [40]. As described in the introduction, following other studies that measured behavioral intentions or adoption, we used the construct as an implementation outcome [23]. For all measures, the physicians could respond to items on a five-point Likert scale.

### Measurements: covariates

Physician characteristics and structural factors were included as covariates. Physician characteristics included age, gender, and work experience in ambulatory care in full years. Age was categorized into three groups (< 50 years, > 50 years, and > 60 years). Gender was dichotomized into male and female (because no answer was provided in the “diverse” category). Structural factors included the position within the primary care organization (i.e., practice owner or employee) and the regional location of the primary care organization (located in a rural or urban area).

## Results

### Qualitative data

The initial qualitative data collection of the evaluation study was conducted with 27 physicians, of whom 15 were in the intervention group and 12 were in the waitlist control group. A brief summary of the qualitative findings is provided as an overview; details of the qualitative data collection and the COREQ checklist used have been published elsewhere [43, 44].

Different behavior-related outcomes were identified: sensitization to risks related to polypharmacy; perceived changes of interdisciplinary and doctor–patient cooperation and communication; and learning effects through using the digital tool. The findings from the two RCT arms were similar in terms of physicians’ awareness of high-risk prescription scenarios with polypharmacy and reflections on changes in professional responsibilities when using digital support for decision making. Qualitative findings were synthesized to describe three different scenarios of simple and complex pathways, which have been differentiated paradigmatically with increasing complexity. The main findings of the qualitative study were captured in the qualitative model, and three relevant themes (prescription safety, information quality, and communication) were selected to operationalize the construct of contextual beliefs, which we predicted would have a significant impact on the main mechanism in the mediation model.

### Descriptive statistics

Three hundred nineteen physicians who fulfilled the inclusion criteria were contacted. The final sample for our SEM research model included 179 physicians with complete data in all variables of interest, out of a total of 218 physicians (response rate of 68%) (see Table 1). The vast majority of participating physicians in the study were men (65%) between 50 and 60 years of age. The participants had an average working experience of 17 years (see Table 1). The study population represents the potentially includable population of primary care physicians in the region where the intervention was implemented in terms of the distribution of gender and age. The patient enrollment ratio averaged 59.97, indicating that physicians used the application for polypharmacy management for an average of 60% of the proposed patients.

**Table 1:** Descriptive statistics

Physician survey respondents (*n* = 218)	
	Mean or proportion of sample
**Sociodemographic variables**	
Gender	
**Female**	35%
**Male**	65%
Age	
**< 50 (y)**	29%
**50–60 (y)**	46%
**> 60 (y)**	25%
Physician work experience (y)	17.03 (SD: 9.11)
**Structural variables**	
Practice type	
**Practice owner**	92%
**Employee physician**	8%
Practice location (region)	
**< 10.000 (i)**	23%
**> 10.000 (i)**	19%
**> 20.000 (i)**	34%
**> 100.000 (i)**	24%

*y* years, *SD* standard deviation, *i* = inhabitants

### Psychometric properties of the measurement analysis

To test for unidimensionality, exploratory factor analyses of the individual construct items and their Cronbach alpha reliabilities were first examined. The results of these analyses revealed that all scale items associated with a given construct or subconstruct loaded highly (> 0.70) on a single factor. One item from the behavioral intention to use construct violated this threshold slightly (0.56), and it also demonstrated loadings on a subconstruct of the contextualized beliefs, although these were weak (0.27). As a result, the final items were analyzed in the measurement model (MM) using CFA. Validation of the MM was performed by examining discriminant and convergent validity and reliability. The results indicated that the values for factor loadings and average variance extracted (AVE) were above recommended thresholds (> 0.5), with the value of the context-specific construct lying slightly below the cutoff. The composite reliability of each factor was above the threshold of 0.7, as were the internal reliability values (Cronbach’s alpha > 0.7). Discriminant validity was assessed by calculating squared correlations of latent variables with any other latent constructs. Discriminant validity was assumed only if all AVE values were greater than square correlations of latent variables. Recommended cutoff values for fit indices supporting MM used in SEM are presented in Additional File 5.

### Structural equation modeling: hypothesis testing

**Fig. 3:**
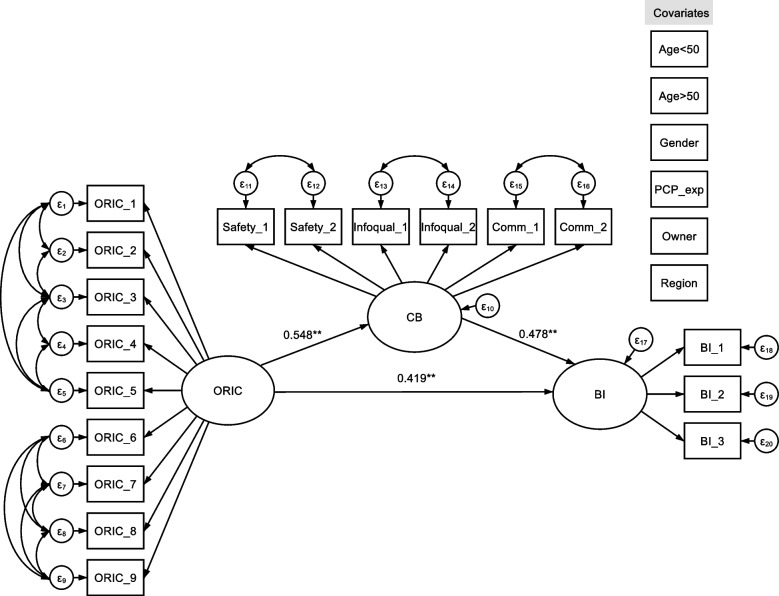
Structural equation model. Note: See Table 2 for all effect measures; see Additional File 5 for all factor loadings of the measurable indicators on the latent constructs. Error terms omitted for visualization purposes. ORIC = organizational readiness for implementing change; CB = contextualized innovation effectiveness beliefs; BI = behavioral intention/adoption; PCP exp = primary care physicians’ working experience (years)

The results presented in Table 2 correspond to the SEM in Fig. 3 and meet the requirements for mediation analysis. Organizational readiness for change is significantly associated with behavioral intention to use innovation (c-path) and physicians’ contextualized beliefs (a-path), and contextualized innovation effectiveness beliefs are significantly associated with behavioral intentions (b-path). The table compares the main statistical measures with and without the addition of covariates. After including the covariates, the effect measures changed slightly, with the largest difference in the b-path. In total, 65% of variance in BI is explained by CBs and ORIC (*R*^2^ = 0.645).

**Table 2:** Standardized estimates of structural equation modeling

Path (a – c)	Direct effect	Indirect effect	Total effect
**Path a:**			
Organizational Readiness (ORIC) – > Contextualized beliefs (CB)	0.560** [CI: 0.25 – 0.69]	N/A	0.560** [CI: 0.25 – 0.69]
Std. Error	0.112		0.112
**Path a (with covariates):**			
Organizational Readiness (ORIC) – > Contextualized beliefs (CB)	0.548** [CI: 0.24 – 0.64]	N/A	0.548** [CI: 0.24 – 0.64]
Std. Error	0.103		0.103
**Path b:**			
Contextualized beliefs (CB) – > Behavioral Intention (BI)	0.510** [CI: 0.23 – 0.90]	N/A	0.510** [CI: 0.23 – 0.90]
Std. Error	0.169		0.169
**Path b (with covariates):**			
Contextualized beliefs (CB) – > Behavioral Intention (BI)	0.478** [CI: 0.25 – 0.92]	N/A	0.478** [CI: 0.25 – 0.92]
Std. Error	0.170		0.170
**Path c:**			
Organizational Readiness (ORIC) – > Behavioral Intention (BI)	0.388** [CI: 0.14 – 0.58]	0.286** [CI: 0.10 – 0.43]	0.674** [CI: 0.48 – 0.78]
Std. Error	0.114	0.082	0.077
**Path c (with covariates):**			
Organizational Readiness (ORIC) – > Behavioral Intention (BI)	0.419** [CI: 0.18 – 0.65]	0.262** [CI: 0.10 – 0.41]	0.681** [CI: 0.49 – 0.87]
Std. Error	0.118	0.079	0.097
**Covariates**			
Age < 50^a^	-0.075	-0.031	-0.106
Age > 50^a^	0.006	0.047	0.054
Gender	-0.054	0.017	-0.036
Length of PCP experience (Years)	0.004	0.027	0.031
Structural: Practice owner^b^	-0.040	-0.007	-0.048
Structural: Urban areac (GP practice)	0.068	0.068	0.137*

Model Fit Statistics: CFI = 0.985; TLI = 0.981; RMSEA = 0.034.; SRMR = 0.055 (with covariates)

Total effects is the sum of direct and indirect effects; Indirect effects are the product of the regression coefficient leading to the outcome. For example, for CB, ORIC predicts CB and CB predicts BI. The indirect effect equals the product of the two regression coefficients from path a * path b

Effects of covariates relate to M and Y

^**^Statistically Significant (*p* < 0.01)

^*^ Statistically Significant (*p* < 0.05)

^a^Compared to Age > 60

^b^Compared to employed primary care physicans (PCP)

^c^Compared to rural area

Overall, the results demonstrate that the hypotheses (H1–H4) and the path model can be validated as a result of good global and local fit indices (CFI = 0.985, RMSEA = 0.034). From the results, it can be inferred that the proportion of the total effect for the outcome behavioral intention or adoption mediated by physicians’ contextualized beliefs is 0.38%, and the ratio of the indirect effect to the direct effect is 0.62, or approximately $\frac{3}{5}$ of the direct effect (H1). Physician and structural characteristics had no effect despite the regional variable (H5).

## Discussion

Our overarching research objective was to examine complex implementation mechanisms that may explain variations in behavior-related outcomes, such as the adoption of innovation. To this end, we examined the relations between the three meso- and micro-level factors, organizational readiness for change, contextualized innovation effectiveness beliefs, and adoption. The findings indicate that an organizationally triggered mechanism lead to the adoption of innovation. The study further demonstrates how contextualized innovation beliefs function within the mechanism, which contributes to a broader understanding of the mechanism.

The empirical findings of this study contribute to the literature on realist evaluation research, organizational change, and the study of implementation mechanisms [45, 46]. The notion that physicians’ beliefs about the expected effective impact of digital innovation is critical to their intentions to adopt innovations is supported by the mediation effect highlighted in this study. Physicians’ perceptions of the effectiveness of digital innovation can actually enhance the impact of organizational readiness for change on innovation adoption. The inference from the indirect effect further supports that implementation strategies of primary care organizations that are not currently using digital innovations for polypharmacy management would benefit from addressing physicians' beliefs about the effectiveness of the innovation [46].

Regarding the last hypothesis, the control factors had no direct or indirect effects and only the regional variable exhibited minor effects. The results indicate that the implementation of digital innovation in urban or rural physician practices is significant. We can derive one possible explanation for this from our qualitative data collection; compared to physicians from rural areas, physicians from urban areas report that they are less knowledgeable about the medical histories and medications of some of their patients, because they see some patients only briefly. Therefore, it is more likely that they will expect additional benefit from using a digital innovation that provides patient-relevant information.

In this paper, we presented a novel approach to SEM mediation analysis that integrates qualitative findings from a context-mechanism-outcome configuration (realist inquiry) and a data-driven latent construct. In addition, the synergistic effects of the two analytic approaches of realist evaluation and SEM were explored. To the best of our knowledge and compared to previous studies in the field of polypharmacy management research [47–49], this study is the first to address the underlying mechanisms in the change process of implementing digital innovation for polypharmacy management in primary care. We achieved a broader understanding of the processes and relations between the micro and meso levels and the effects on physician adoption behavior.

The current state of research on polypharmacy indicates that there is an urgent worldwide need to simplify the complex clinical practice of polypharmacy management, because an increasing number of elderly and multimorbid patients will be affected by polypharmacy, and the workload of primary care physicians who care for these patients with complex medication regimens will increase [1, 47]. Hence, digital solutions that meet the physicians’ needs and are regarded by them as effective tools for patient care are being sought.

### Practical implications

At the time of data collection, the physicians were using digital innovation for approximately 60% of the patients who could potentially derive benefit from it. This indicates that the application was not yet fully implemented. This observation may have practical implications for developing implementation strategies for respondents who have not used digital innovation actively, have only used it infrequently, or have not even begun implementing it. Insights into the specific beliefs about the effectiveness of the innovation allow inferences to refine the initial qualitative model of the configuration of context, mechanism, and outcome (see Fig. 1) based on the empirical findings. A middle-range theory of the main complex implementation mechanism in our study setting may be described as follows:

The employees of primary health care organizations should be encouraged to develop a high readiness for change and to be prepared to perform new tasks. Innovation developers must understand which topics are relevant from the physicians’ perspective, so that the physicians will perceive innovation as effective and adopt it (mechanism [reasoning] → outcome). Physicians need digital innovation that sensitizes them to the risks of polypharmacy, creates a learning effect, and provides valuable and helpful information for practice (mechanism [resources]). Beyond that, digital innovation must serve to reassure and support clinicians in ambiguous decision and communication situations with polypharmacy patients, when (de-) prescribing medications (context → mechanism [reasoning]). Organizations or researchers can use these findings to adapt primary care digital innovation and implementation strategies to improve digital health technology adoption (context → mechanism [reasoning + resources] → outcome) for polypharmacy management [50].

### Strengths and limitations

The sample consisted of primary care physicians who implemented digital innovation for polypharmacy management. Only physicians who had participated in the study between 2018 and 2020 and were part of the intervention group during that period were included in the data analyses. It is likely that physicians recruited for the study at a later date had different initial conditions, because technical problems had been resolved and better communication strategies had been developed. Non-participation in the survey could be ascribed to physicians not using digital innovation regularly at the time of data collection and, therefore, being unable to provide responses.

The inferences drawn from the two strands of qualitative and quantitative data analysis were merged to create a comprehensive understanding of the digital innovation implementation process. The application of the modified methodological approach in this study enabled us to integrate the interdisciplinary evidence on the topic of contextual influences on change processes through realist evaluation. We then explored the intersection with a quantitative analysis method that uses a theory-based confirmatory approach to examine statistical relationships (SEM) [12].

The methodological synergy effects are particularly reflected in the development of the contextual measurement instrument, in which the findings of the qualitative content analysis and CMO were integrated. Furthermore, the directions of the effects and relationships of the micro and meso levels and the corresponding measurement instruments were determined on this basis and confirmed in the mediation model. As explained in the previous section, our analyses indicate reasonable reliability as well as the convergent and discriminant validity of the measurement instruments used in this study. In addition, the requirements for mediation analysis were met. Therefore, we argue that our study provides a robust methodological basis to confirm semi-predictable patterns between contexts, underlying generative mechanisms, and outcomes in primary care settings. Future studies should plan their study design accordingly to conduct more advanced, strictly quantitative mediation analyses. In the present study design, the focus was on the triangulation of the different models in the two study phases. To minimize bias, we sequentially analyzed qualitative and quantitative data and confirmed the assumptions made in the first study phase with the findings of the second phase [33]. In addition, we emphasized the methodological approach of realist inquiry and interpreted the theory-building assumptions in this framework.

## Conclusion

Implementation research indicates inconsistent implementation effectiveness, possibly related to the proximal outcomes of the actual implementation behavior during the change processes. This study explored the underlying mechanisms. Empirical confirmation of contextual mechanisms expands the theories regarding the functioning of mechanisms triggered by the implementation of digital health technology. In addition, this study confirms that organizational readiness for change has a direct effect on physician adoption behavior. However, this relationship is indirectly affected by individual beliefs regarding the effectiveness of the innovation.

The adoption behavior of primary care physicians correlates strongly with the degree of meso-level readiness to implement change, as well as with the extent to which physicians view the digital innovation as beneficial to their work. Innovation beliefs are related to three subdimensions that pertain to the extent to which the use of digital innovations is perceived as effective: (1) to improve patient safety, (2) to improve clinical decision-making during the course of risk and interaction analysis, and (3) to improve communication regarding the management of polypharmacy for patients in the context of ambiguous decision situations.

To the best of our knowledge, this is the first study to provide new insights into in-depth local needs assessment. The adoption of digital innovations for polypharmacy management in primary care organizations can be improved by tailoring implementation strategies accordingly. Our findings contribute to the understanding of the underlying mechanisms and complex adaptive processes of social systems that operate in a primary care setting. Therefore, our approach provides methodological insights into realist evaluation and contributes to current research that seeks to illustrate the complex contextual pathways and their effect on implementation outcomes [10, 11].

## Supplementary Information

**Additional file 1.** Flow diagram of the study.

**Additional file 2.** Study protocol section of the formative evaluation study.

**Additional file 3.** Interview guide.

**Additional file 4.** Contextualized innovation effectiveness beliefs scale (CB).

**Additional file 5.** Construct reliability of measuring instruments.

**Additional file 6.** AdAM Study Group.

## Data Availability

The datasets generated and analyzed during the current study are not publicly available due to participant consent restricting data use to the research team but are available from the corresponding author on reasonable request.

## Abbreviations

AdAM: Application of a digitally assisted pharmacotherapy management system (original German acronym for the c-RCT project)
AVE: Average variance extracted
BI: Behavioral intention to use/adoption
CB: Contextualized innovation effectiveness beliefs
CFI: Comparative fit index
CFIR: Consolidated framework of implementation research
CMOc: Context – mechanism – outcome configuration
c-RCT: Cluster-randomized controlled trial
IT: Information technology
MM: Measurement model
ORIC: Organizational readiness for implementing change
RMSEA: Root mean square error of approximation
SEM: Structural equation model
TAM: Technology acceptance model
TLI: Tucker – Lewis index
TPB: Theory of planned behavior
TRA: Theory of reasoned action

## References

[1] Feldman SS, Buchalter S, Hayes LW (2018). Health Information Technology in Healthcare Quality and Patient Safety: Literature Review. JMIR Med Inform.

[2] Lainer M, Mann E, Sönnichsen A (2013). Information technology interventions to improve medication safety in primary care: a systematic review. Int J Qual Health Care.

[3] Jaspers MWM, Smeulers M, Vermeulen H (2011). Effects of clinical decision-support systems on practitioner performance and patient outcomes: A synthesis of high-quality systematic review findings. J Am Med Inform Assoc.

[4] Lainer M (2013). Information technology interventions to improve medication safety in primary care: A systematic review. Int J Qual Health Care..

[5] Ammenwerth E, Schnell-Inderst P, Machan C (2008). The effect of electronic prescribing on medication errors and adverse drug events: a systematic review. J Am Med Informatics Assoc JAMIA.

[6] van de Velde S, Heselmans A, Delvaux N (2018). A systematic review of trials evaluating success factors of interventions with computerised clinical decision support. Implement Sci.

[7] Garavand A, Mohseni M, Asadi H (2016). Factors influencing the adoption of health information technologies: A systematic review. Electron Physician.

[8] Rieckert A, Teichmann AL, Drewelow E (2019). Reduction of inappropriate medication in older populations by electronic decision support (the PRIMA-eDS project): a survey of general practitioners' experiences. J Am Med Inform Assoc.

[9] Chau PYK, Hu PJH (2001). Information Technology Acceptance by Individual Professionals: A Model Comparison Approach*. Decis Sci..

[10] Greenhalgh J, Manzano A (2022). Understanding ‘context’ in realist evaluation and synthesis. Int J Soc Res Methodol.

[11] Dalkin SM, Greenhalgh J, Jones D (2015). What's in a mechanism? Development of a key concept in realist evaluation. Implementation Sci.

[12] Brown A, Hecker KG, Bok H (2021). Strange bedfellows: exploring methodological intersections between realist inquiry and structural equation modeling. J Mixed Methods Res.

[13] Moore GF, Evans RE (2017). What theory, for whom and in which context? Reflections on the application of theory in the development and evaluation of complex population health interventions. SSM Popul Health.

[14] Damschroder LJ, Aron DC, Keith RE (2009). Fostering implementation of health services research findings into practice: a consolidated framework for advancing implementation science. Implementation Sci.

[15] Damschroder LJ, Reardon CM, Widerquist MAO (2022). The updated Consolidated Framework for Implementation Research based on user feedback. Implementation Sci.

[16] Li S-A, Jeffs L, Barwick M (2018). Organizational contextual features that influence the implementation of evidence-based practices across healthcare settings: a systematic integrative review. Syst Rev.

[17] Paré G, Sicotte C, Poba-Nzaou P (2011). Clinicians' perceptions of organizational readiness for change in the context of clinical information system projects: insights from two cross-sectional surveys. Implementation Sci.

[18] Weiner BJ (2009). A theory of organizational readiness for change. Implement Sci.

[19] Klein KJ, Conn AB, Sorra JS (2001). Implementing computerized technology: an organizational analysis. J Appl Psychol.

[20] Mallidou AA, Atherton P, Chan L (2018). Core knowledge translation competencies: a scoping review. BMC Health Serv Res.

[21] Godin G (2008). Healthcare professionals' intentions and behaviours: A systematic review of studies based on social cognitive theories. Implement Sci.

[22] Holden RJ, Karsh B-T (2010). The technology acceptance model: Its past and its future in health care. J Biomed Inform.

[23] Proctor E, Silmere H, Raghavan R (2011). Outcomes for implementation research: conceptual distinctions, measurement challenges, and research agenda. Adm Policy Ment Health.

[24] Eccles MP, Hrisos S, Francis J (2006). Do self- reported intentions predict clinicians' behaviour: a systematic review. Implementation Sci.

[25] Sheeran P (2002). Intention—behavior relations: a conceptual and empirical review. Eur Rev Soc Psychol.

[26] Miller A, Moon B, Anders S (2015). Integrating computerized clinical decision support systems into clinical work: a meta-synthesis of qualitative research. Int J Med Inform.

[27] Kouri A, Yamada J, Lam Shin Cheung J (2022). Do providers use computerized clinical decision support systems? A systematic review and meta-regression of clinical decision support uptake. Implementation Sci.

[28] May CR, Johnson M, Finch T (2016). Implementation, context and complexity. Implementation Sci.

[29] Pfadenhauer LM, Gerhardus A, Mozygemba K (2017). Making sense of complexity in context and implementation: the Context and Implementation of Complex Interventions (CICI) framework. Implementation Sci.

[30] Müller BS, Klaaßen-Mielke R, Gonzalez-Gonzalez AI (2021). Effectiveness of the application of an electronic medication management support system in patients with polypharmacy in general practice: a study protocol of cluster-randomised controlled trial (AdAM). BMJ Open.

[31] Dillman DA, Smyth JD, Christian LM. Internet, phone, mail, and mixed-mode surveys: the tailored design method. Wiley; 2014.

[32] Cameron R (2009). A sequential mixed model research design: Design, analytical and display issues. International Journal of Multiple Research Approaches.

[33] Creswell JW, Clark VLP. Designing and conducting mixed methods research. Sage Publications; 2017.

[34] Hsieh H-F, Shannon SE (2005). Three approaches to qualitative content analysis. Qual Health Res.

[35] Aichholzer J (2017). Einführung in lineare Strukturgleichungsmodelle mit Stata.

[36] Kline RB. Principles and practice of structural equation modeling. Guilford Publications; 2023.

[37] Koufteros XA (1999). Testing a model of pull production: a paradigm for manufacturing research using structural equation modeling. J Oper Manag.

[38] Hu L, Bentler PM (1999). Cutoff criteria for fit indexes in covariance structure analysis: Conventional criteria versus new alternatives. Struct Equ Modeling.

[39] Shea CM, Jacobs SR, Esserman DA, et al. Organizational readiness for implementing change: a psychometric assessment of a new measure. Implementation Sci. 2014;9(1):1–15. 10.1186/1748-5908-9-7 PMC3904699 24410955

[40] Abdekhoda M, Ahmadi M, Gohari M (2015). The effects of organizational contextual factors on physicians' attitude toward adoption of Electronic Medical Records. J Biomed Inform.

[41] Weiner BJ, Amick H, Lee S-YD (2008). Conceptualization and measurement of organizational readiness for change: a review of the literature in health services research and other fields. Med Care Res Rev.

[42] Lindig A, Hahlweg P, Christalle E (2020). Translation and psychometric evaluation of the German version of the Organisational Readiness for Implementing Change measure (ORIC): a cross-sectional study. BMJ Open.

[43] Tong A, Sainsbury P, Craig J (2007). Consolidated criteria for reporting qualitative research (COREQ): a 32-item checklist for interviews and focus groups. Int J Qual Health Care.

[44] Söling S, Köberlein-Neu J, Müller BS (2020). From sensitization to adoption? A qualitative study of the implementation of a digitally supported intervention for clinical decision making in polypharmacy. Implement Sci.

[45] Lewis CC, Boyd MR, Walsh-Bailey C (2020). A systematic review of empirical studies examining mechanisms of implementation in health. Implementation Sci.

[46] Geng EH, Baumann AA, Powell BJ (2022). Mechanism mapping to advance research on implementation strategies. PLoS Med.

[47] Molokhia M, Majeed A (2017). Current and future perspectives on the management of polypharmacy. BMC Fam Pract.

[48] McIntosh J, Alonso A, MacLure K (2018). A case study of polypharmacy management in nine European countries: Implications for change management and implementation. PLoS One.

[49] Kurczewska-Michalak M, Lewek P, Jankowska-Polańska B (2021). Polypharmacy Management in the Older Adults: A Scoping Review of Available Interventions. Front Pharmacol.

[50] Powell BJ, Waltz TJ, Chinman MJ (2015). A refined compilation of implementation strategies: results from the Expert Recommendations for Implementing Change (ERIC) project. Implementation Sci.

