# Supplementary figures and images for: Complex implementation mechanisms in primary care: do physicians’ beliefs about the effectiveness of innovation play a mediating role? Applying a realist inquiry and structural equation modeling approach in a formative evaluation study
Source: BMC Prim Care. 2023 Jun 27;24:131. doi: 10.1186/s12875-023-02081-x (PMC10294464; doi:10.1186/s12875-023-02081-x)

Additional File 1. Flow diagram of the study

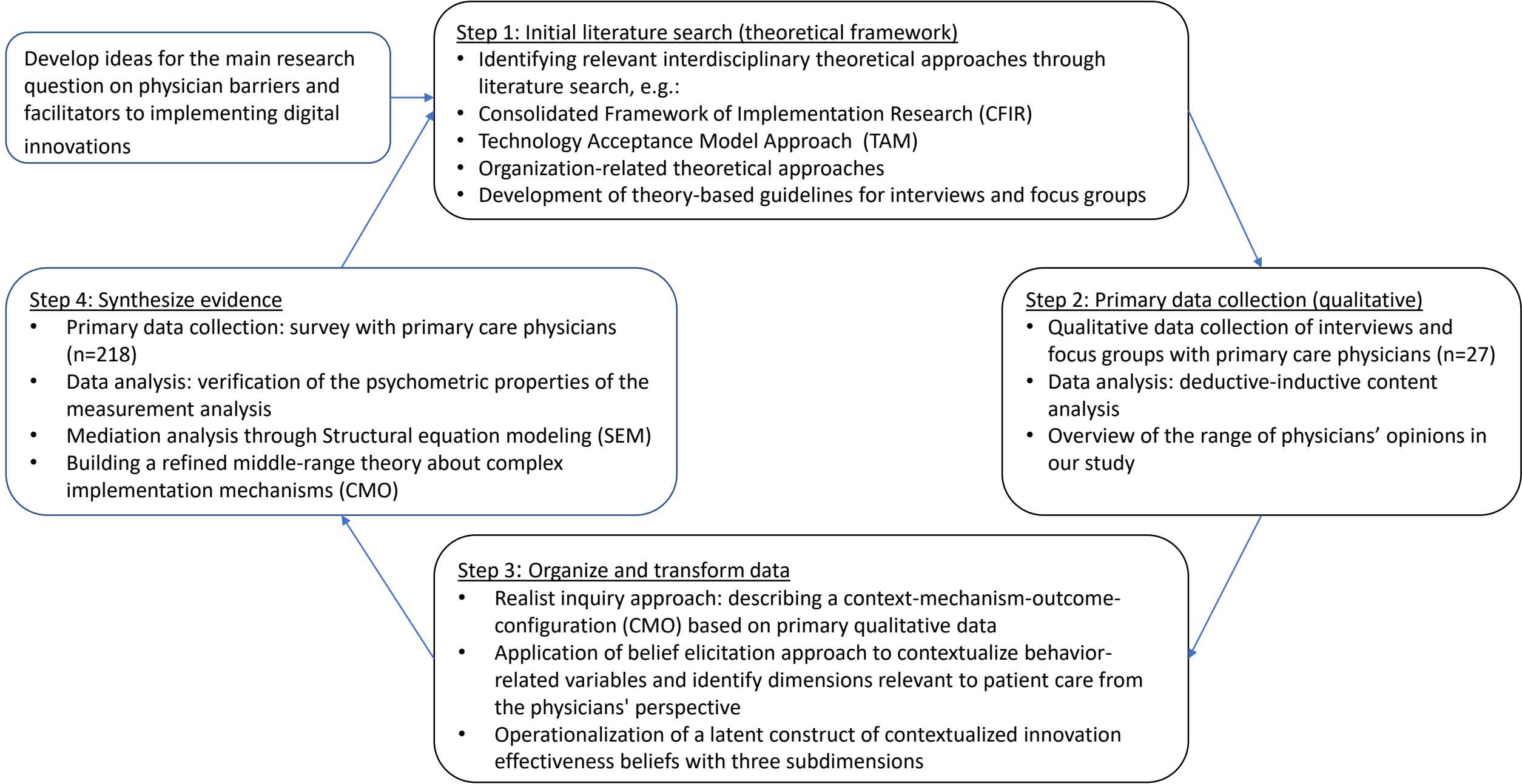

Supplement: Supplementary file 1 — Additional file 1. Flow diagram of the study. [file 12875_2023_2081_MOESM1_ESM.pdf]
