# Supplementary material for: Complex implementation mechanisms in primary care: do physicians’ beliefs about the effectiveness of innovation play a mediating role? Applying a realist inquiry and structural equation modeling approach in a formative evaluation study
Source: BMC Prim Care. 2023 Jun 27;24:131. doi: 10.1186/s12875-023-02081-x (PMC10294464; doi:10.1186/s12875-023-02081-x)
Supplement: Supplementary file 2 — Additional file 2. Study protocol section of the formative evaluation study. [file 12875_2023_2081_MOESM2_ESM.pdf]

Additional File 2

Study protocol section of the formative evaluation study. Analysis of barriers and facilitators: Qualitative Interviews, Focus Groups and Survey with Physicians, in:

Müller, B. S., Klaaßen-Mielke, R., Gonzalez-Gonzalez, A. I., Grandt, D., Hammerschmidt, R., Köberlein-Neu, J., ... & Muth, C. (2021). Effectiveness of the application of an electronic medication management support system in patients with polypharmacy in general practice: a study protocol of cluster-randomised controlled trial (AdAM). BMJ open, 11(9), e048191.

The aim of this sub-study is to identify factors facilitating or hindering the successful implementation of the intervention from a general practitioner's point of view and evaluate which factors facilitate or hinder the effective performance of systematic medication-checks and optimization. Hereby is expected to get insights how the intervention can be optimized and adapted for general practitioners' high-level acceptance and effectiveness of optimized medication-checks by area-wide implementation. Therefore, a multistage mixed-methods-Approach will be conducted (combination of qualitative and quantitative outcomes) (1).

Level 1: To analyze general practitioners subjectively perceived barriers and resources regarding implementation, guided expert-interviews will be conducted (n= 5-10) (face-to-face-interviews or telephone-interviews) (2,3) to explore the field. Therefore, a convenient sample strategy will be applied. Furthermore, formative evaluation will take part during the trial with two additional time points of qualitative data collection related to relevant emerging topics concerning successful implementation.

Level 2: Results of qualitative data collection will be used for understanding practical orientation patterns of general practitioners (how do they actually use AdAM in real life settings) and their conjunctive experiential space (4). Focus groups with general practitioners of intervention and control group (total, n= 4) will be conducted concerning their experiences and expectations of the project.

Level 3: Results of qualitative data collection will be used to prepare a quantitative general practitioners survey, in which all participating physicians of the intervention group will be asked about barriers and facilitators of the implementation. The survey aims representative detection of general practitioners factors, which facilitate or hinder implementation and identify specific attributes of 'early adapters' and 'late adapters' (5). Quantitative data will be evaluated descriptive and by applying appropriate multiple regression models.

The quality of the qualitative research data collection and analysis in interviews and focus groups is assured by audio recording as well as by transcription according to established standards and by independent coding and subsequent interpretation by a group of researchers. Data analysis will comprise qualitative content analysis according to Kuckartz (6). Quality assurance concerning the survey conduct is assured by standards of survey development, pretesting, Dillman's Total Design (7) method for increasing response rates and data preparation with the Teleform® software.

REFERENCES

1. Mayring P. Evidenztriangulation in der Gesundheitsforschung. KZfSS Kölner Zeitschrift für Soziologie und Sozialpsychologie. 2017 Oct 10;69(S2):415–34.
2. Bogner A, Littig B, Menz W. Experteninterviews. Theorien, Methoden, Anwendungsfelder. 3., grundlegend überarbeitete Auflage. Wiesbaden: VS Verlag für Sozialwissenschaft; 2009.
3. Christmann G. Telefonische Experteninterviews. In: Alexander Bogner, Beate Littig und Wolfgang Menz (Hg): Experteninterviews Theorien, Methoden, Anwendungsfelder 3, grundlegend überarbeitete Auflage. Wiesbaden: VS Verlag für Sozialwissenschaften; 2009.
4. Bohnsack R. Dokumentarische Methode und sozialwissenschaftliche Hermeneutik. Zeitschrift für Erziehungswiss. 2003;6(4):550–70.
5. Rogers EM. Diffusion of preventive innovations. Addict Behav. 2002 Nov;27(6):989–93.
6. Kuckartz U. Einführung in die computergestützte Analyse qualitativer Daten. Wiesbaden: VS Verlag für Sozialwissenschaften; 2007.
7. Dillman DA, Smmyth JS, Christian LM. Internet, phone, mail, and mixed-mode surveys: the tailored design method. Wiley; 2014. 528 p.
