# Supplementary material for: Complex implementation mechanisms in primary care: do physicians’ beliefs about the effectiveness of innovation play a mediating role? Applying a realist inquiry and structural equation modeling approach in a formative evaluation study
Source: BMC Prim Care. 2023 Jun 27;24:131. doi: 10.1186/s12875-023-02081-x (PMC10294464; doi:10.1186/s12875-023-02081-x)
Supplement: Supplementary file 3 — Additional file 3. Interview guide. [file 12875_2023_2081_MOESM3_ESM.pdf]

Additional File 3. Interview Guide.

Themes	Key question	Follow-up question
Polypharmacy care situation	Ath the beginning of the interview, I would like you to ask you to tell me how good patient care can be provided to multimorbid patients with polypharmacy.	a) Please describe a situation with a patient in which the patient's multimedication care was well managed.<br>b) Please explain what you think were the reasons you were able to provide good care.
	Please describe what poor quality patient care looks like for multimorbid patients with polypharmacy.	a) Please describe a situation with a patient in which the patient's multimedication care was less well managed.<br>b) Please explain what you think were the reasons you were not able to provide good care.<br>c) What are some other barriers to good patient care?
	How do you document multimedication in your practice or what is documented?	a) Please describe how the documentation of a patient's multimedication is done in your practice and who is involved (e.g., practice staff) and how the documentation is done (e.g., by computer, by hand).
AdAM	We are particularly interested in the implementation of AdAM in your practice.<br>Please tell us about the extent to which you have been able to integrate AdAM into your daily work with the resources available in your practice.	a) How do you think AdAM can improve the care situation?<br>b) What motivated you to participate in AdAM and what were your expectations for the project?<br>c) To what extent did your practice's technical equipment influence your decision to participate?<br>d) To what extent did the per-patient payment affect participation?
	Please share what topics you have discussed with your recent AdAM patients.	a) How did you explain AdAM to your patients and how did they accept it?<br>b) How do you discuss prescribing new medications or discontinuing medications with your patients in AdAM and how do they respond?
	We are interested in how AdAM has changed the framework of care for multimorbid patients in your practice. Please describe to what extent AdAM has contributed to the change in the care situation.	a) To what extent do you use pharmaceutical information provided by AdAM to make decisions about prescribing or discontinuing medications?<br>b) To what extent do you use the ability to share information about your patient's co-treatment with physician colleagues that AdAM provides to you when making decisions about prescriptions or discontinuing medications?<br>c) How do you handle recommendations and alerts from AdAM?<br>d) In your opinion, to what extent should AdAM be changed to improve patient care?
At the end of the interview, I would like to ask you if there is anything on this topic in general that is important from your point of view.		
