# Supplementary material for: Complex implementation mechanisms in primary care: do physicians’ beliefs about the effectiveness of innovation play a mediating role? Applying a realist inquiry and structural equation modeling approach in a formative evaluation study
Source: BMC Prim Care. 2023 Jun 27;24:131. doi: 10.1186/s12875-023-02081-x (PMC10294464; doi:10.1186/s12875-023-02081-x)
Supplement: Supplementary file 4 — Additional file 4. Contextualized innovation effectiveness beliefs scale (CB). [file 12875_2023_2081_MOESM4_ESM.pdf]

Additional File 4

Contextualized innovation effectiveness beliefs scale (CB)

Item no.		Dimension
1	I think using the [name of digital innovation] has made me more aware of the risks of polypharmacy.	
2	I think that by using [name of digital innovation], I will be able to transfer my newly acquired knowledge about polypharmacy to other patients in this primary care organization.	*Prescription Safety*
	What information about your polypharmacy patients provided by [digital innovation name] has been most helpful to you in patient care?	*Information Quality*
3	a. Risk analysis information	
4	b. Side effect analysis information	
5	Using [name of digital innovation] in our primary care organization improves communication with my polypharmacy patients.	*Communication*
6	By using [name of digital innovation] in our primary care organization, I can better explain to my polypharmacy patients why their medications should be discontinued or switched.	

Note: For all measures, physicians were able to respond to items on a five-point Likert scale.
