# Supplementary material for: Complex implementation mechanisms in primary care: do physicians’ beliefs about the effectiveness of innovation play a mediating role? Applying a realist inquiry and structural equation modeling approach in a formative evaluation study
Source: BMC Prim Care. 2023 Jun 27;24:131. doi: 10.1186/s12875-023-02081-x (PMC10294464; doi:10.1186/s12875-023-02081-x)
Supplement: Supplementary file 5 — Additional file 5. Construct reliability of measuring instruments. [file 12875_2023_2081_MOESM5_ESM.pdf]

Additional File 5. Construct reliability of measuring instruments

Constructs	Mean (SD)	AVE	CR	Items	Factor Loading
Organizational Readiness for Implementing Change (ORIC)	3.17 (1.03)	0.759	0.967		
				ORIC1	.790
				ORIC2	.828
				ORIC3	.854
				ORIC4	.874
				ORIC5	.870
				ORIC6	.814
				ORIC7	.807
				ORIC8	.830
				ORIC9	.885
Contextualized innovation effectiveness beliefs (CB)	3.36 (.94)	0.490	0.842		
				CB1	.545
				CB2	.648
				CB3	.624
				CB4	.614
				CB5	.708
				CB6	.757
Behavioral Intention (BI)/ Adoption	2.43 (.91)	0.620	0.832		
				BI1	.808
				BI2	.809
				BI3	.741

For all measures physicians could respond items on a five-point Likert scale from 1 (strongly disagree) to 5 (strongly agree).
