# Supplementary material for: Complex implementation mechanisms in primary care: do physicians’ beliefs about the effectiveness of innovation play a mediating role? Applying a realist inquiry and structural equation modeling approach in a formative evaluation study
Source: BMC Prim Care. 2023 Jun 27;24:131. doi: 10.1186/s12875-023-02081-x (PMC10294464; doi:10.1186/s12875-023-02081-x)
Supplement: Supplementary file 6 — Additional file 6. AdAM Study Group. [file 12875_2023_2081_MOESM6_ESM.pdf]

Additional File 6

AdAM Study Group

Petra Kellermann-Mühlhoff[4], (AdAM Study Group representative; Petra.Kellermann-Muehlhoff@barmer.de), Lara Düvel[4], Till Beckmann[4], Reinhard Hammerschmidt[5], Julia Jachmich[5], Eva Leicher[5], Benjamin Brandt[5], Johanna Richard[5], Frank Meyer[5], Dr. Mathias Flume[5], Thomas Müller[5], Prof Dr. Ferdinand M. Gerlach[6], Prof. Dr. Christiane Muth[6], Dr. Ana Isabel Gonzalez-Gonzalez[6], Kiran Chapidi[6], Robin Brünn[6], Peter Ihle[7], Ingo Meyer[7], Prof. Dr. Nina Timmesfeld[8], Prof. Dr. Hans J. Trampisch[8], Renate Klaaßen-Mielke[8], Jale Basten[8], Prof. Dr. Wolfgang Greiner[9], Bastian Suhrmann[9], Alexandra Piotrowski[10], Karolina Beifus[10], Sarah Meyer[10], Prof. Dr. Daniel Grandt[11], Simone Grandt[12]

[4] BARMER, Statutory Health Insurance, Wuppertal, Germany

[5] KVWL, Association of statutory health insurance physicians Westfalen-Lippe, Dortmund, Germany

[6] Institute of General Practice, Goethe-University, Frankfurt/Main, Germany

[7] PMV research group, Faculty of Medicine and University Hospital Cologne, University of Cologne, Cologne, Germany

[8] Department of Medical Informatics, Biometry and Epidemiology, Ruhr University, Bochum, Germany

[9]Faculty of Health Science, Department of Health Economics and Health Care Management, Bielefeld University, Bielefeld, Germany

[10]Center for Health Economics and Health Services Research, University of Wuppertal, Germany

[11] Internal Medicine I, at the Clinic Saarbrücken, Germany

[12] RpDoc Solutions GmbH, Saarbrücken, Germany
